# dl-Methyl 4-(4-meth­oxy­phen­yl)-2,7,7-trimethyl-5-oxo-1,4,5,6,7,8-hexa­hydro­quinoline-3-carboxyl­ate

**DOI:** 10.1107/S1600536812005892

**Published:** 2012-02-17

**Authors:** Jing-Min Zhao

**Affiliations:** a Institute of Higher Vocational Education, Tongliao Vocational College, Inner Mongolia Autonomous Region Tongliao, Huolinhe Street No. 152, 028000, People’s Republic of China

## Abstract

In the title compound, C_21_H_25_NO_4_, the dihydropyridine ring adopts a flattened boat conformation. The N atom and the *sp* ^3^ C atom deviate in the same direction from the mean plane of the other four C atoms, by 0.269 (6) and 0.111 (6) Å, respectively. This mean plane is inclined to the 4-methoxy­phenyl ring by 87.3 (5)°. The cyclohexenone ring has a sofa conformation with the C atom bearing the methyl groups deviating from the mean plane through the other five C atoms by 0.628 (6) Å. There is a short C—H⋯O hydrogen bond in the molecule. In the crystal, molecules are linked by an N—H⋯O hydrogen bond to form chains propagating along the *c*-axis direction.

## Related literature

For related structures and hydrogen-bond definition, see: Yang *et al.* (2010 ▶). For the syntheis method, see: Tamaddon *et al.* (2010 ▶); Yang *et al.* (2011 ▶). For related literature about the biological activity of 1,4-dihydropyridines and their derivatives, see: Davies *et al.* (2005 ▶); Rose & Draeger (1992 ▶); Warrior *et al.* (2005 ▶).
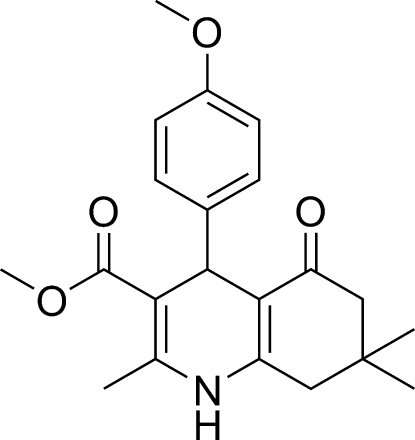


## Experimental

### 

#### Crystal data

C_21_H_25_NO_4_
*M* *_r_* = 355.42
Tetragonal, 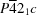

*a* = 16.058 (2) Å
*c* = 14.343 (3) Å
*V* = 3698.5 (11) Å^3^
*Z* = 8
Mo *K*α radiation
μ = 0.09 mm^−1^
*T* = 293 K
0.20 × 0.10 × 0.10 mm

#### Data collection

Nonius CAD-4 diffractometer
Absorption correction: ψ scan (North *et al.*, 1968 ▶). *T*_min_ = 0.983, *T*_max_ = 0.991
6166 measured reflections
3353 independent reflections
1856 reflections with *I* > 2σ(*I*)
*R*_int_ = 0.069
3 standard reflections every 200 reflections intensity decay: 1%

#### Refinement

*R*[*F* ^2^ > 2σ(*F* ^2^)] = 0.066
*wR*(*F* ^2^) = 0.161
*S* = 1.01
3353 reflections
235 parameters
H-atom parameters constrained
Δρ_max_ = 0.21 e Å^−3^
Δρ_min_ = −0.19 e Å^−3^

### 

Data collection: *CAD-4 EXPRESS* (Enraf–Nonius, 1994 ▶); cell refinement: *CAD-4 EXPRESS*; data reduction: *XCAD4* (Harms & Wocadlo, 1996 ▶); program(s) used to solve structure: *SHELXS97* (Sheldrick, 2008 ▶); program(s) used to refine structure: *SHELXL97* (Sheldrick, 2008 ▶); molecular graphics: *SHELXTL* (Sheldrick, 2008 ▶); software used to prepare material for publication: *SHELXTL*.

## Supplementary Material

Crystal structure: contains datablock(s) I, global. DOI: 10.1107/S1600536812005892/ld2046sup1.cif

Structure factors: contains datablock(s) I. DOI: 10.1107/S1600536812005892/ld2046Isup2.hkl

Supplementary material file. DOI: 10.1107/S1600536812005892/ld2046Isup3.mol

Supplementary material file. DOI: 10.1107/S1600536812005892/ld2046Isup4.cml

Additional supplementary materials:  crystallographic information; 3D view; checkCIF report

## Figures and Tables

**Table 1:** Hydrogen-bond geometry (Å, °)

*D*—H⋯*A*	*D*—H	H⋯*A*	*D*⋯*A*	*D*—H⋯*A*
N—H0*A*⋯O1^i^	0.86	2.02	2.868 (4)	169
C12—H12*A*⋯O3	0.96	2.17	2.895 (6)	131

Symmetry code: (i) 
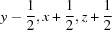
.

**Fig. 1.**
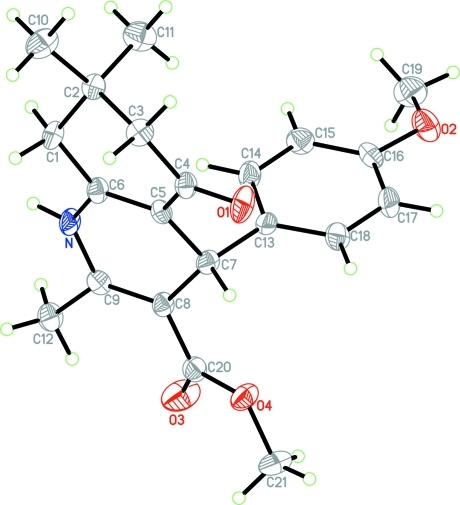
Molecular structure of the title compound, showing the atom-numbering scheme. Displacement ellipsoids are drawn at the 50% probability level.

**Fig. 2.**
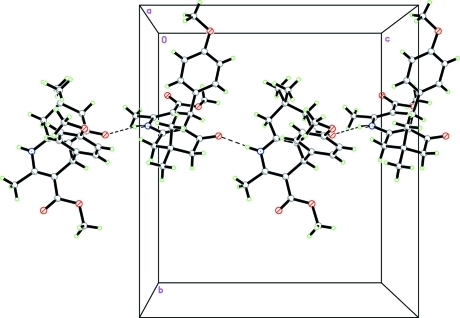
The packing of the title compound, viewed along the a axis. Dashed lines indicate hydrogen bonds.

## supplementary crystallographic information

### Comment

1,4-Dihydropyridines and their derivatives are an important class of pharmaceutical compounds with a broad spectrum of biological activities. For example, they have calcium modulatory properties (Rose & Draeger 1992), antibacterial (Davies *et al.* 2005), fungicidal (Warrior *et al.* 2005), antioxidant activities (Yang *et al.* 2011) *etc*. Therefore, significant interest has been attracted to find out convenient and facile approaches for the synthesis of 1,4-dihydropyridines. In view of the exhibited biological activitiy, precise single-crystal structure determinations of these derivatives are expected to provide insights in their design and function.

### Experimental

The title compound was obtained according to the reported method (Tamaddon *et al.*, 2010). A mixture of 4-Methoxybenzaldehyde (2 mmol), methyl acetoacetate (2 mmol), 5,5-dimethylcyclohexane-1,3-dione (2 mmol) and NH_4_HCO_3_ (2 mmol) was stirred in water (2 ml) under reflux. After completion of the reaction (TLC monitoring), the mixture was diluted with cold water (20 ml) and filtered to obtain the precipitated product which was further purified by recrystallization. Single crystals suitable for X-ray diffraction were obtained by slow evaporation of an ethanol solution. IR (KBr) *v*/cm^-1^: 3181, 3067, 2960, 1703, 1604; ^1^H NMR (300 MHz, DMSO-*d6*) *δ*/p.p.m.: 9.07 (s, 1H, NH), 7.04(d, 2H, ArH, J = 8.4 Hz), 6.74 (d, 2H, ArH, J = 8.4 Hz), 4.80 (s, 1H, H4), 3.67, 3.53 (2 s, 6H, 2OCH_3_), 1.92–2.51 (m, 7H, cyclohexaneone), 1.00, 0.84 (2 s, 6H, 2CH_3_); MS (ESI) m/*z*: 378.2 [*M*+Na]^+^, 394.2 [*M*+K]^+^

### Refinement

All H atoms were located in a difference map and refined isotropically. The N—H distance was constrained to 0.86 Å. All other H atoms were positioned geometrically and treated as riding, with C—H distances in the range 0.93–0.96 Å, and *U*_iso_(H) = 1.2 or 1.5 times *U*_eq_(C). The methyl groups were allowed to rotate during the refinement.

### Crystal data

**Table:**

C_21_H_25_NO_4_	*D*_x_ = 1.277 Mg m^−^^3^
*M**_r_* = 355.42	Mo *K*α radiation, λ = 0.71073 Å
Tetragonal, *P*42_1_*c*	Cell parameters from 25 reflections
Hall symbol: P -4 2n	θ = 9–12°
*a* = 16.058 (2) Å	µ = 0.09 mm^−^^1^
*c* = 14.343 (3) Å	*T* = 293 K
*V* = 3698.5 (11) Å^3^	Block, light yellow
*Z* = 8	0.20 × 0.10 × 0.10 mm
*F*(000) = 1520	

### Data collection

**Table:**

Nonius CAD-4 diffractometer	1856 reflections with *I* > 2σ(*I*)
Radiation source: fine-focus sealed tube	*R*_int_ = 0.069
Graphite monochromator	θ_max_ = 25.4°, θ_min_ = 1.8°
ω/2θ scans	*h* = 0→19
Absorption correction: ψ scan For Semi-empirical (using intensity measurements) absorption, see: (North *et al.*, 1968).	*k* = −10→19
*T*_min_ = 0.983, *T*_max_ = 0.991	*l* = 0→17
6166 measured reflections	3 standard reflections every 200 reflections
3353 independent reflections	intensity decay: 1%

### Refinement

**Table:**

Refinement on *F*^2^	Primary atom site location: structure-invariant direct methods
Least-squares matrix: full	Secondary atom site location: difference Fourier map
*R*[*F*^2^ > 2σ(*F*^2^)] = 0.066	Hydrogen site location: inferred from neighbouring sites
*wR*(*F*^2^) = 0.161	H-atom parameters constrained
*S* = 1.01	*w* = 1/[σ^2^(*F*_o_^2^) + (0.060*P*)^2^] where *P* = (*F*_o_^2^ + 2*F*_c_^2^)/3
3353 reflections	(Δ/σ)_max_ < 0.001
235 parameters	Δρ_max_ = 0.21 e Å^−^^3^
0 restraints	Δρ_min_ = −0.19 e Å^−^^3^

### Special details

**Table:**

Geometry. All e.s.d.'s (except the e.s.d. in the dihedral angle between two l.s. planes) are estimated using the full covariance matrix. The cell e.s.d.'s are taken into account individually in the estimation of e.s.d.'s in distances, angles and torsion angles; correlations between e.s.d.'s in cell parameters are only used when they are defined by crystal symmetry. An approximate (isotropic) treatment of cell e.s.d.'s is used for estimating e.s.d.'s involving l.s. planes.
Refinement. Refinement of *F*^2^ against ALL reflections. The weighted *R*-factor *wR* and goodness of fit *S* are based on *F*^2^, conventional *R*-factors *R* are based on *F*, with *F* set to zero for negative *F*^2^. The threshold expression of *F*^2^ > σ(*F*^2^) is used only for calculating *R*-factors(gt) *etc*. and is not relevant to the choice of reflections for refinement. *R*-factors based on *F*^2^ are statistically about twice as large as those based on *F*, and *R*- factors based on ALL data will be even larger.

### Fractional atomic coordinates and isotropic or equivalent isotropic displacement parameters (Å^2^)

**Table:**

	*x*	*y*	*z*	*U*_iso_*/*U*_eq_	
N	0.4727 (3)	0.8718 (2)	0.5450 (2)	0.0371 (11)	
H0A	0.4565	0.8757	0.6020	0.044*	
O1	0.4081 (2)	0.9125 (2)	0.2288 (2)	0.0550 (10)	
C1	0.3498 (3)	0.9515 (3)	0.5073 (3)	0.0404 (13)	
H1A	0.3695	1.0068	0.5234	0.049*	
H1B	0.3265	0.9267	0.5631	0.049*	
O2	0.3890 (3)	0.5017 (2)	0.2656 (3)	0.0583 (10)	
C2	0.2815 (3)	0.9593 (3)	0.4341 (3)	0.0344 (12)	
O3	0.6966 (2)	0.7414 (3)	0.4707 (3)	0.0709 (13)	
C3	0.3222 (3)	0.9807 (3)	0.3409 (3)	0.0406 (13)	
H3A	0.2799	0.9793	0.2926	0.049*	
H3B	0.3435	1.0371	0.3441	0.049*	
O4	0.6735 (2)	0.7892 (2)	0.3268 (2)	0.0474 (10)	
C4	0.3928 (3)	0.9230 (3)	0.3128 (3)	0.0357 (13)	
C5	0.4399 (3)	0.8842 (3)	0.3850 (3)	0.0313 (12)	
C6	0.4217 (3)	0.9006 (3)	0.4758 (3)	0.0339 (12)	
C7	0.5083 (3)	0.8241 (3)	0.3582 (3)	0.0353 (12)	
H7A	0.5378	0.8467	0.3040	0.042*	
C8	0.5700 (3)	0.8157 (3)	0.4383 (3)	0.0331 (12)	
C9	0.5500 (3)	0.8365 (3)	0.5264 (3)	0.0343 (12)	
C10	0.2205 (3)	1.0282 (3)	0.4621 (4)	0.0581 (16)	
H10A	0.1952	1.0145	0.5207	0.087*	
H10B	0.1781	1.0334	0.4152	0.087*	
H10C	0.2499	1.0800	0.4678	0.087*	
C11	0.2338 (4)	0.8785 (3)	0.4250 (4)	0.0589 (16)	
H11A	0.2715	0.8346	0.4079	0.088*	
H11B	0.1918	0.8843	0.3779	0.088*	
H11C	0.2080	0.8652	0.4835	0.088*	
C12	0.6027 (3)	0.8290 (3)	0.6124 (3)	0.0445 (14)	
H12A	0.6556	0.8053	0.5962	0.067*	
H12B	0.5752	0.7938	0.6568	0.067*	
H12C	0.6111	0.8832	0.6391	0.067*	
C13	0.4736 (3)	0.7388 (3)	0.3322 (3)	0.0339 (12)	
C14	0.4306 (3)	0.6932 (3)	0.3972 (3)	0.0427 (14)	
H14A	0.4208	0.7165	0.4555	0.051*	
C15	0.4013 (3)	0.6140 (3)	0.3791 (3)	0.0455 (14)	
H15A	0.3729	0.5843	0.4248	0.055*	
C16	0.4147 (3)	0.5795 (3)	0.2921 (3)	0.0402 (13)	
C17	0.4554 (3)	0.6252 (3)	0.2247 (3)	0.0458 (14)	
H17A	0.4630	0.6028	0.1655	0.055*	
C18	0.4852 (3)	0.7040 (3)	0.2445 (3)	0.0442 (14)	
H18A	0.5133	0.7339	0.1987	0.053*	
C19	0.3666 (4)	0.4456 (3)	0.3372 (4)	0.0629 (18)	
H19A	0.3491	0.3938	0.3101	0.094*	
H19B	0.3217	0.4688	0.3730	0.094*	
H19C	0.4137	0.4361	0.3770	0.094*	
C20	0.6523 (3)	0.7785 (3)	0.4173 (3)	0.0394 (13)	
C21	0.7556 (3)	0.7591 (4)	0.3010 (4)	0.0569 (16)	
H21A	0.7653	0.7700	0.2361	0.085*	
H21B	0.7589	0.7003	0.3123	0.085*	
H21C	0.7970	0.7872	0.3376	0.085*	

### Atomic displacement parameters (Å^2^)

**Table:**

	*U*^11^	*U*^22^	*U*^33^	*U*^12^	*U*^13^	*U*^23^
N	0.046 (3)	0.048 (3)	0.0169 (19)	0.002 (2)	−0.0004 (18)	−0.0006 (19)
O1	0.081 (3)	0.063 (3)	0.0214 (16)	0.020 (2)	0.0000 (19)	0.0002 (18)
C1	0.049 (3)	0.041 (3)	0.032 (3)	0.008 (3)	0.005 (2)	−0.006 (2)
O2	0.072 (3)	0.044 (2)	0.060 (2)	−0.017 (2)	−0.007 (2)	−0.004 (2)
C2	0.040 (3)	0.027 (3)	0.036 (3)	0.005 (3)	0.000 (3)	0.003 (2)
O3	0.051 (3)	0.109 (4)	0.052 (2)	0.027 (2)	−0.004 (2)	0.023 (3)
C3	0.045 (3)	0.033 (3)	0.043 (3)	0.005 (3)	0.000 (3)	0.008 (3)
O4	0.044 (2)	0.062 (2)	0.036 (2)	0.007 (2)	0.0093 (17)	0.0025 (19)
C4	0.045 (3)	0.034 (3)	0.027 (3)	0.000 (3)	−0.001 (2)	0.001 (2)
C5	0.035 (3)	0.031 (3)	0.029 (3)	0.000 (2)	−0.007 (2)	0.000 (2)
C6	0.045 (3)	0.032 (3)	0.025 (2)	−0.003 (3)	0.001 (2)	0.002 (2)
C7	0.035 (3)	0.049 (3)	0.022 (2)	0.003 (3)	−0.001 (2)	0.003 (2)
C8	0.040 (3)	0.035 (3)	0.024 (2)	−0.003 (2)	0.001 (2)	0.002 (2)
C9	0.040 (3)	0.034 (3)	0.029 (3)	−0.008 (3)	−0.004 (2)	0.007 (2)
C10	0.054 (4)	0.066 (4)	0.054 (4)	0.013 (3)	0.007 (3)	0.008 (3)
C11	0.056 (4)	0.063 (4)	0.058 (4)	−0.006 (3)	−0.003 (3)	0.011 (3)
C12	0.050 (4)	0.053 (3)	0.031 (3)	0.001 (3)	−0.007 (3)	0.002 (2)
C13	0.030 (3)	0.043 (3)	0.028 (3)	0.001 (2)	0.000 (2)	−0.005 (2)
C14	0.053 (4)	0.047 (4)	0.028 (3)	−0.004 (3)	0.004 (3)	−0.004 (3)
C15	0.047 (4)	0.046 (4)	0.043 (3)	−0.008 (3)	0.003 (3)	0.006 (3)
C16	0.039 (3)	0.036 (3)	0.046 (3)	−0.005 (3)	−0.012 (3)	−0.004 (3)
C17	0.059 (4)	0.049 (4)	0.029 (3)	−0.004 (3)	−0.005 (3)	−0.007 (3)
C18	0.049 (3)	0.058 (4)	0.026 (3)	−0.004 (3)	−0.004 (2)	0.001 (3)
C19	0.063 (4)	0.045 (4)	0.081 (4)	−0.007 (3)	0.013 (4)	0.002 (3)
C20	0.042 (3)	0.043 (3)	0.033 (3)	−0.006 (3)	0.002 (3)	0.000 (3)
C21	0.037 (3)	0.073 (4)	0.061 (4)	0.007 (3)	0.014 (3)	0.006 (3)

### Geometric parameters (Å, º)

**Table:**

N—C6	1.368 (6)	C9—C12	1.501 (6)
N—C9	1.389 (6)	C10—H10A	0.9600
N—H0A	0.8600	C10—H10B	0.9600
O1—C4	1.240 (5)	C10—H10C	0.9600
C1—C6	1.485 (7)	C11—H11A	0.9600
C1—C2	1.523 (6)	C11—H11B	0.9600
C1—H1A	0.9700	C11—H11C	0.9600
C1—H1B	0.9700	C12—H12A	0.9600
O2—C16	1.370 (6)	C12—H12B	0.9600
O2—C19	1.412 (6)	C12—H12C	0.9600
C2—C11	1.512 (7)	C13—C14	1.371 (6)
C2—C3	1.528 (6)	C13—C18	1.390 (6)
C2—C10	1.532 (6)	C14—C15	1.380 (7)
O3—C20	1.203 (5)	C14—H14A	0.9300
C3—C4	1.518 (6)	C15—C16	1.382 (6)
C3—H3A	0.9700	C15—H15A	0.9300
C3—H3B	0.9700	C16—C17	1.379 (7)
O4—C20	1.352 (5)	C17—C18	1.382 (7)
O4—C21	1.453 (5)	C17—H17A	0.9300
C4—C5	1.427 (6)	C18—H18A	0.9300
C5—C6	1.360 (6)	C19—H19A	0.9600
C5—C7	1.512 (7)	C19—H19B	0.9600
C7—C8	1.523 (6)	C19—H19C	0.9600
C7—C13	1.524 (7)	C21—H21A	0.9600
C7—H7A	0.9800	C21—H21B	0.9600
C8—C9	1.346 (6)	C21—H21C	0.9600
C8—C20	1.482 (7)		
			
C6—N—C9	122.2 (4)	H10A—C10—H10C	109.5
C6—N—H0A	118.9	H10B—C10—H10C	109.5
C9—N—H0A	118.9	C2—C11—H11A	109.5
C6—C1—C2	113.3 (4)	C2—C11—H11B	109.5
C6—C1—H1A	108.9	H11A—C11—H11B	109.5
C2—C1—H1A	108.9	C2—C11—H11C	109.5
C6—C1—H1B	108.9	H11A—C11—H11C	109.5
C2—C1—H1B	108.9	H11B—C11—H11C	109.5
H1A—C1—H1B	107.7	C9—C12—H12A	109.5
C16—O2—C19	117.2 (4)	C9—C12—H12B	109.5
C11—C2—C1	110.7 (4)	H12A—C12—H12B	109.5
C11—C2—C3	109.5 (4)	C9—C12—H12C	109.5
C1—C2—C3	108.2 (4)	H12A—C12—H12C	109.5
C11—C2—C10	108.6 (4)	H12B—C12—H12C	109.5
C1—C2—C10	109.9 (4)	C14—C13—C18	117.9 (5)
C3—C2—C10	109.9 (4)	C14—C13—C7	119.8 (4)
C4—C3—C2	114.5 (4)	C18—C13—C7	122.2 (4)
C4—C3—H3A	108.6	C13—C14—C15	122.4 (5)
C2—C3—H3A	108.6	C13—C14—H14A	118.8
C4—C3—H3B	108.6	C15—C14—H14A	118.8
C2—C3—H3B	108.6	C14—C15—C16	119.1 (5)
H3A—C3—H3B	107.6	C14—C15—H15A	120.4
C20—O4—C21	115.5 (4)	C16—C15—H15A	120.4
O1—C4—C5	122.7 (5)	O2—C16—C17	115.7 (5)
O1—C4—C3	119.3 (4)	O2—C16—C15	124.7 (5)
C5—C4—C3	118.0 (4)	C17—C16—C15	119.5 (5)
C6—C5—C4	119.8 (4)	C18—C17—C16	120.5 (5)
C6—C5—C7	121.6 (4)	C18—C17—H17A	119.7
C4—C5—C7	118.6 (4)	C16—C17—H17A	119.7
C5—C6—N	120.0 (4)	C17—C18—C13	120.5 (5)
C5—C6—C1	124.4 (4)	C17—C18—H18A	119.8
N—C6—C1	115.5 (4)	C13—C18—H18A	119.8
C5—C7—C8	109.7 (4)	O2—C19—H19A	109.5
C5—C7—C13	111.8 (4)	O2—C19—H19B	109.5
C8—C7—C13	110.0 (4)	H19A—C19—H19B	109.5
C5—C7—H7A	108.4	O2—C19—H19C	109.5
C8—C7—H7A	108.4	H19A—C19—H19C	109.5
C13—C7—H7A	108.4	H19B—C19—H19C	109.5
C9—C8—C20	120.2 (4)	O3—C20—O4	121.7 (5)
C9—C8—C7	122.1 (4)	O3—C20—C8	126.7 (4)
C20—C8—C7	117.6 (4)	O4—C20—C8	111.5 (4)
C8—C9—N	119.6 (4)	O4—C21—H21A	109.5
C8—C9—C12	128.1 (5)	O4—C21—H21B	109.5
N—C9—C12	112.3 (4)	H21A—C21—H21B	109.5
C2—C10—H10A	109.5	O4—C21—H21C	109.5
C2—C10—H10B	109.5	H21A—C21—H21C	109.5
H10A—C10—H10B	109.5	H21B—C21—H21C	109.5
C2—C10—H10C	109.5		
			
C6—C1—C2—C11	73.4 (6)	C20—C8—C9—N	179.7 (4)
C6—C1—C2—C3	−46.7 (5)	C7—C8—C9—N	−4.2 (7)
C6—C1—C2—C10	−166.7 (4)	C20—C8—C9—C12	1.6 (8)
C11—C2—C3—C4	−69.0 (5)	C7—C8—C9—C12	177.7 (5)
C1—C2—C3—C4	51.8 (5)	C6—N—C9—C8	−12.4 (7)
C10—C2—C3—C4	171.8 (4)	C6—N—C9—C12	166.0 (4)
C2—C3—C4—O1	152.0 (5)	C5—C7—C13—C14	−61.7 (6)
C2—C3—C4—C5	−29.1 (6)	C8—C7—C13—C14	60.4 (6)
O1—C4—C5—C6	177.8 (5)	C5—C7—C13—C18	119.6 (5)
C3—C4—C5—C6	−1.0 (7)	C8—C7—C13—C18	−118.3 (5)
O1—C4—C5—C7	−3.5 (8)	C18—C13—C14—C15	2.0 (8)
C3—C4—C5—C7	177.7 (4)	C7—C13—C14—C15	−176.8 (5)
C4—C5—C6—N	−172.0 (4)	C13—C14—C15—C16	−0.8 (8)
C7—C5—C6—N	9.3 (7)	C19—O2—C16—C17	164.3 (5)
C4—C5—C6—C1	5.6 (8)	C19—O2—C16—C15	−16.6 (8)
C7—C5—C6—C1	−173.0 (5)	C14—C15—C16—O2	179.8 (5)
C9—N—C6—C5	9.8 (7)	C14—C15—C16—C17	−1.2 (8)
C9—N—C6—C1	−168.1 (4)	O2—C16—C17—C18	−178.9 (5)
C2—C1—C6—C5	20.1 (7)	C15—C16—C17—C18	2.0 (8)
C2—C1—C6—N	−162.1 (4)	C16—C17—C18—C13	−0.8 (8)
C6—C5—C7—C8	−22.5 (6)	C14—C13—C18—C17	−1.2 (8)
C4—C5—C7—C8	158.8 (4)	C7—C13—C18—C17	177.6 (5)
C6—C5—C7—C13	99.8 (5)	C21—O4—C20—O3	−4.9 (7)
C4—C5—C7—C13	−78.9 (5)	C21—O4—C20—C8	176.4 (4)
C5—C7—C8—C9	20.0 (6)	C9—C8—C20—O3	23.6 (8)
C13—C7—C8—C9	−103.3 (5)	C7—C8—C20—O3	−152.7 (5)
C5—C7—C8—C20	−163.7 (4)	C9—C8—C20—O4	−157.8 (5)
C13—C7—C8—C20	72.9 (5)	C7—C8—C20—O4	25.9 (6)

### Hydrogen-bond geometry (Å, º)

**Table:**

*D*—H···*A*	*D*—H	H···*A*	*D*···*A*	*D*—H···*A*
N—H0*A*···O1^i^	0.86	2.02	2.868 (4)	169
C12—H12*A*···O3	0.96	2.17	2.895 (6)	131

Symmetry code: (i) *y*−1/2, *x*+1/2, *z*+1/2.
